# Short-Term Physiological Effects of Red Blood Cell Transfusion in Very Low Birth Weight Infants: A Retrospective Cohort Study

**DOI:** 10.3390/children13060830

**Published:** 2026-06-18

**Authors:** Charlotte Aßmann, Philipp Deindl, Martin E. Blohm, Dominique Singer, Ahmed Aboalqez

**Affiliations:** 1 Division of Neonatology and Pediatric Intensive Care Medicine, University Children’s Hospital, University Medical Center Hamburg-Eppendorf, 20246 Hamburg, Germany; 2 Department of Pediatrics and Adolescent Medicine, Hospital of Lüneburg, 21339 Lüneburg, Germany; 3 Department of Neonatology and Pediatric Intensive Care Medicine, Altona Children´s Hospital, 22763 Hamburg, Germany

**Keywords:** very-low-birth-weight infants, packed red blood cell transfusion, intermittent hypoxemia, bradycardia, non-invasive respiratory support, cardiorespiratory stability, neonatal intensive care, short-term physiological effects

## Abstract

**Highlights:**

**What are the main findings?**

- Packed red blood cell (PRBC) transfusion in non-intubated very-low-birth-weight infants was associated with a significant reduction in frequency of bradycardia episodes within 24 h after transfusion.
- PRBC transfusion reduced the depth of oxygen desaturation events without significantly altering FiO_2_ requirements and baseline heart rate.

**What is the implication of the main findings?**

- PRBC transfusion may selectively improve cardiorespiratory function in the short term in clinically stable preterm infants.

**Abstract:**

**Background/Objectives**: While packed red blood cell transfusions are commonly administered in anemic neonates, transfusion strategies in preterm infants have been the subject of debate for decades, particularly due to questionable long-term benefits and limited evidence regarding short-term physiological effects. In non-intubated preterm infants, established transfusion thresholds are considered, but individual clinical judgment often plays an important role in the final decision. This study aims to assess the short-term cardiorespiratory effects of red blood cell transfusions in non-intubated very-low-birth-weight (VLBW) infants who were either spontaneously breathing or receiving non-invasive respiratory support. **Methods**: Retrospective, single-center analysis of 68 VLBW infants (<1500 g) who received 99 red blood cell transfusions between 2019 and 2023. Cardiorespiratory parameters were observed over a 24 h period before and after transfusion. **Results**: Following transfusion, there was a significant decrease in the frequency of bradycardia events per 24 h (6.51 ± 5.55 to 4.24 ± 3.8; *p* = 0.004), accompanied by an improvement in the depth of oxygen desaturations (78.7 ± 4.18 to 81.0 ± 3.71; *p* = 0.001). No significant changes were detected in the desaturation frequency, FiO_2_ or heart rate. **Conclusions**: In clinically stable very-low-birth-weight infants receiving non-invasive ventilatory support, packed red blood cell transfusion is associated with modest, short-term improvements in cardiorespiratory stability. However, these effects are limited in scope. Further research is needed to identify which patient subgroups derive the most significant benefit from these transfusions.

## 1. Introduction

Very-low-birth-weight (VLBW) preterm infants frequently experience apnea, bradycardia and intermittent oxygen desaturation due to immature respiratory regulation [1]. Anemia is a common and clinically relevant condition in this population and has been associated with increased cardiorespiratory instability, including desaturation and bradycardia episodes [2,3]. Packed red blood cell (PRBC) transfusions are commonly used to manage this instability [4].

Transfusion practice in neonatology has been the subject of study for many decades, evolving from predominantly liberal approaches targeting higher hemoglobin levels towards more restrictive strategies as evidence from clinical trials accumulated [3,4,5,6,7]. This shift was reinforced by evidence of risks associated with donor exposure and transfusion-related adverse effects, as well as by randomized trial data demonstrating non-inferiority regarding outcome in premature infants comparing restrictive versus liberal transfusion strategies [6,7]. The ETTNO trial and TOP trial are presently regarded as the most influential studies in this field and form the basis for restrictive protocols in many neonatal units [6,7]. Despite the implementation of restrictive protocols, transfusion decisions in practice remain largely individualized, especially when cardiorespiratory instability occurs near borderline hemoglobin levels. This persistent uncertainty reflects a gap between defined transfusion thresholds and the physiological variability observed in daily care, particularly in non-intubated infants whose respiratory drive and autonomic regulation differ substantially from critically ill, mechanically ventilated cohorts. The systematic review by Bellach et al. highlights inconsistencies in current transfusion strategies and identifies important gaps in short-term outcome research [8]. While long-term outcomes such as neurodevelopment and bronchopulmonary dysplasia have been extensively studied, short-term physiological responses remain underexplored [9,10,11,12]. Existing studies are limited by heterogeneous definitions of instability, differences in monitoring strategies, and mixed study populations that combine intubated with spontaneously breathing infants, thereby reducing the applicability of their findings to stable, non-intubated preterm infants [13,14]. From a physiological standpoint, anemia and transfusion may influence autonomic stability, myocardial performance, and systemic oxygen delivery [13,15]. However, it remains unclear whether transfusions in clinically stable, non-invasively supported VLBW infants lead to measurable short-term changes in the frequency or depth of cardiorespiratory events. The evidence gap described by Bellach et al. is therefore both conceptual and methodological, as many prior studies do not provide high-resolution analyses of narrow time windows before and after transfusion [8]. This study aims to address this gap by evaluating short-term cardiorespiratory effects of PRBC transfusion in a well-defined cohort of non-intubated VLBW infants using continuous electronic monitoring and standardized event definitions.

## 2. Materials and Methods

This retrospective, observational, single-center study enrolled preterm infants with a birth weight of less than 1500 g who were born between 2019 and 2023 at the University Medical Center Hamburg-Eppendorf. From a total of 389 infants who initially met the inclusion criteria, only those who received at least one PRBC transfusion during their stay in the neonatal intensive care unit (NICU) were considered for analysis. A total of 228 PRBC transfusions were administered to the 68 infants, and 99 of these satisfied all inclusion criteria and were consequently included in the study.

Exclusion criteria included mortality within the observation period, the presence of cyanotic congenital heart disease or complex congenital malformations, and the utilization of invasive mechanical ventilation before, during or shortly after the transfusion. Infants were excluded from the study if they received medications known to affect baseline heart rate, such as catecholamines. In order to reduce heterogeneity and isolate short-term physiological effects, the study population was intentionally restricted to clinically stable preterm infants receiving only non-invasive respiratory support, such as spontaneous breathing, continuous positive airway pressure (CPAP) or noninvasive positive pressure ventilation (NIPPV).

In accordance with local clinical protocols, all infants born before 34 weeks gestational age routinely received caffeine citrate at a dose of 10–15 milligrams per kilogram per day. The present study did not analyze the effect of caffeine citrate on cardiorespiratory parameters. Transfusion thresholds at the NICU were based on national guidelines [16] and generally reflect a restrictive transfusion approach in alignment with the ETTNO trial [6]. Accordingly, transfusion triggers followed the restrictive arm of the ETTNO trial and corresponded to hematocrit levels of <28% (day 0–7), <24% (day 8–21), and <21% (day >21). The final decision to transfuse was made by the clinical team, considering the infant’s overall clinical condition, cardiorespiratory stability, and laboratory values. PRBC transfusions were administered at a median volume of 20 mL/kg, in accordance with institutional protocols. To ensure statistical independence of repeated events in infants receiving multiple transfusions, a period of 48 h was defined as the minimum interval between transfusions.

This study focused on analyzing vital parameters in the 24 h preceding and 24 h following each transfusion. In accordance with local NICU standards, the target range for heart rate was 100–200 bpm, and the target SpO_2_ range was 90–100%. The data collection included the frequency and severity of bradycardia, defined as heart rate below 100 beats per minute (bpm), and desaturation episodes, defined as peripheral oxygen saturation (SpO_2_) below 90%, along with the baseline heart rate and the fraction of inspired oxygen (FiO_2_). Clinical responses to instability, such as tactile stimulation, manual positive pressure ventilation or bag-mask ventilation, were documented to capture behavioral and therapeutic interventions triggered by events and included in the analysis.

Patient data, including vital signs and transfusion records, were extracted from the electronic patient data management system (PDMS ICM, Dräger, Lübeck, Germany). Vital signs were continuously monitored via electrocardiography (ECG) and pulse oximetry. To ensure the clinical validity of the captured events and to distinguish true physiological occurrences from artifacts, a combined workflow of automated digital extraction and manual verification was applied. This process effectively reduced classification errors and confirmed adherence to exclusion criteria. Both extraction approaches produced fully concordant datasets.

All collected parameters were statistically analyzed as follows. To assess systematic changes 24 h before and after the transfusion, paired statistical tests were applied. For continuous, interval-scaled variables with an assumed approximate normal distribution (e.g., mean heart rate, mean FiO_2_, mean depth of SpO_2_ desaturations), a paired *t*-test was used. Normality of paired differences was assessed visually (histogram, Q–Q plot) and analytically using the Shapiro–Wilk test. For countable events (e.g., number of bradycardias per 24 h), a paired *t*-test was also performed, provided that the differences between paired observations were approximately normally distributed. In cases where the assumption of normality was violated, the Wilcoxon signed-rank test was used as a non-parametric alternative. The level of significance was set at *p* = 0.05. Statistical analyses were conducted using R (version 4.3.3), supplemented with packages from the tidyverse for data processing and visualization.

## 3. Results

Of the 389 eligible infants, 68 preterm infants received one or more PRBC transfusions and were included in the final analysis. A total of 99 transfusions met all inclusion criteria. Detailed baseline characteristics are summarized in Table 1.

### 3.1. Cardiorespiratory Events

**Figure 1:**
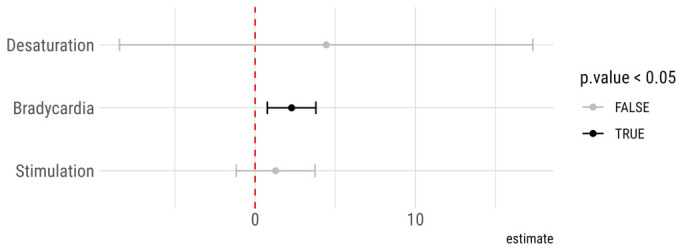
Comparison of documented events before and after the PRBC Transfusion. The plot shows estimated differences in the number of events (post- vs. pre-transfusion) for desaturation, bradycardia, and stimulation. The red dashed line represents blood transfusion.

The analysis of the clinical parameters during the 24 h period before and after transfusions revealed a statistically significant decrease in the number of bradycardia episodes. The mean number of bradycardia events decreased from 6.51 (±5.55) to 4.24 (±3.8) per 24 h, corresponding to a relative reduction of approximately 34.9% (*p* = 0.004). This effect persisted across infants with differing comorbidities, suggesting a consistent short-term stabilizing influence of transfusion on autonomic regulation. Figure 1 displays the estimated differences in documented events for desaturation, bradycardia, and stimulation.

**Figure 2:**
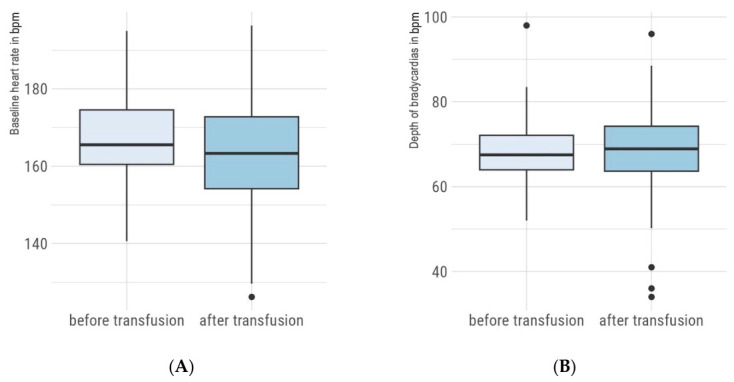
(**A**,**B**) Comparative boxplots illustrating the baseline heart rate (**A**) and minimal heart rate (depth of bradycardias) (**B**) before and after the PRBC transfusion. Both plots display the median, interquartile range (IQR), confidence intervals, and outliers. The baseline heart rate showed no relevant change following transfusion. Similarly, the depth of bradycardias remained consistent across the two time points, indicating no significant change in severity.

The mean baseline heart rate did not show a statistically significant reduction following transfusion (168 bpm ± 9.11 to 164 bpm ± 12.17, *p* = 0.060, 2.4%), as illustrated in Figure 2A. The depth of bradycardia remained unchanged across both time periods, with a mean minimum heart rate of 68.4 bpm (±5.9) before and 68.3 bpm (±9.3) after transfusion. The stable nadir values indicate that transfusion influenced the occurrence but not the severity of bradycardic events. Figure 2B illustrates the overlapping distributions.

### 3.2. Oxygen Desaturation Dynamics

**Figure 3:**
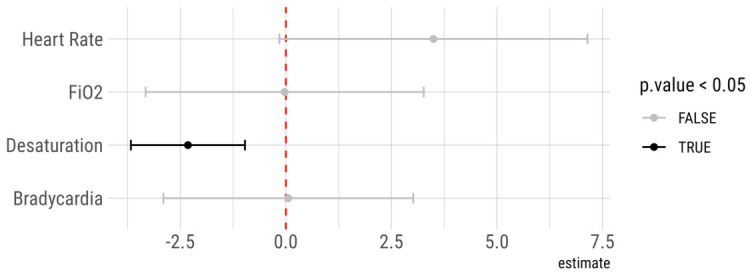
Estimated mean differences in continuous cardiorespiratory parameters before and after PRBC transfusion. Each horizontal line represents one parameter, with points indicating the estimated mean difference and horizontal bars representing the 95% confidence intervals. Positive estimates reflect higher mean values after transfusion, while negative estimates indicate decreases relative to the pre-transfusion period. The red dashed line represents blood transfusion.

**Figure 4:**
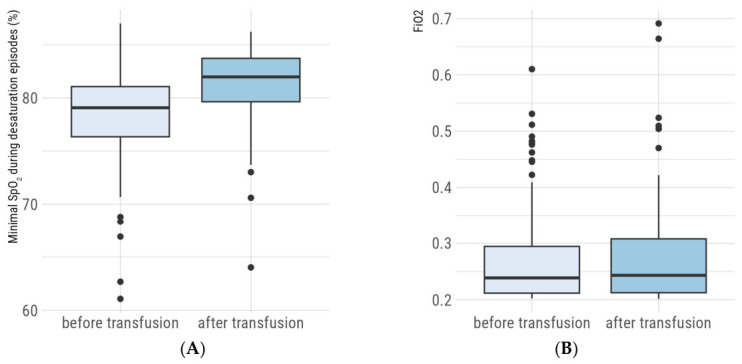
(**A**,**B**) Boxplots illustrating the depth of desaturations (minimal SpO_2_, (**A**)) and oxygen requirement (FiO_2_, right, (**B**)) before and after PRBC transfusion. Each plot displays the median, interquartile range (IQR), confidence intervals, and outliers. The desaturation events were significantly deeper prior to transfusion, indicating more pronounced oxygen drops before intervention. In contrast, FiO_2_ requirements remained unchanged, suggesting that transfusion did not significantly impact baseline oxygen needs.

There was no statistically significant change in the number of desaturation incidents (SpO_2_ < 90%) per 24 h (84.8 ± 46.7 before vs. 80.4 ± 43.5 after; *p* = 0.498), with a relative difference of 5.2% (Figure 1). However, the depth of desaturations exhibited a substantial improvement, with the mean minimum SpO_2_ increasing from 78.7% (±4.18) to 81.0% (±3.17), representing a 2.9% relative increase (*p* = 0.001). This improvement is demonstrated in Figure 3 and Figure 4A.

The supplemental oxygen requirement (FiO_2_) remained unchanged over the observation periods, with a mean of 28.46% (±9.07) before and 28.49% (±9.38) after transfusion (*p* = 0.987). The unchanged FiO_2_ (Figure 4B) suggests that respiratory support requirements were not altered, reinforcing that observed improvements reflect intrinsic physiological responses rather than external adjustments.

### 3.3. Clinical Interventions

Similarly, the number of physical stimulation interventions (tactile stimulation, manual positive pressure ventilation and bag-mask ventilation) showed no significant difference, decreasing slightly from 5.77 (±6.5) to 4.48 (±4.54) per 24 h (*p* = 0.303).

Collectively, these results indicate a selective and physiologically plausible benefit of transfusion, improving stability during events without altering overall respiratory workload or support requirements.

## 4. Discussion

This retrospective single-center study analyzed short-term effects of PRBC transfusion on cardiorespiratory parameters in non-intubated very-low-birth-weight infants. It demonstrated that PRBC transfusion was associated with a significant reduction of 34.9% in the frequency of bradycardia episodes within 24 h after transfusion. PRBC transfusion reduced the depth of oxygen desaturation events without significantly altering fiO_2_ requirements or baseline heart rate.

These effects may alleviate symptoms of cardiorespiratory instability without fundamentally altering respiratory regulation, consistent with earlier findings [2,4,13,14].

The reduction in bradycardia frequency aligns with prior studies linking anemia to autonomic dysregulation [2]. Zagol et al. noted that while transfusions reduced event frequency, severity depended more on intrinsic autonomic mechanisms than on hematologic status alone. The unchanged depth suggests that once initiated, these events tend to follow a course not modified by transfusion [2]. Enhanced myocardial performance post-transfusion may improve the infant’s ability to mitigate physiological stressors, potentially preventing vagally mediated bradycardia, as proposed by Saleemi et al. [15]. Since brief bradycardia episodes (<80 bpm) can impair cerebral oxygenation and reduce cerebral perfusion to levels associated with an increased risk of adverse neurological outcomes, as demonstrated by Pichler et al., reducing their frequency may be clinically significant [17].

A different pattern emerged in the analysis of oxygen desaturation dynamics. While the frequency of events remained constant, their depth decreased significantly, suggesting that PRBC transfusion mitigates severity rather than the occurrence of hypoxic episodes.

Variation in desaturation definitions across studies affects outcomes. Abu Jawdeh et al. used a stricter cutoff (SpO_2_ ≤ 80% for ≥4 s) and observed significant reductions in both frequency and depth following transfusion [18]. Our broader threshold (SpO_2_ < 90%) likely contributed to higher event counts and may have attenuated the measurable post-transfusion effect. The choice of thresholds thus meaningfully shapes both the apparent instability of the cohort and the detectable treatment effect, highlighting the need for standardized outcome definitions in future research.

Even without changes in the frequency of desaturation events, enhancements in desaturation depth may still carry clinical relevance. The BOOST-II and SUPPORT trials have demonstrated that lower oxygen saturation targets are associated with increased mortality and adverse neurodevelopmental outcomes [19,20]. Building upon these findings, Di Fiore et al. have shown that lower saturation targets result in an increased frequency of intermittent hypoxemia, particularly during early and late postnatal periods [1]. This suggests that a cumulative hypoxic burden, rather than individual event severity, may contribute to long-term morbidity. Against this background, even modest improvements in nadir SpO_2_ after transfusion may influence longer-term outcomes by reducing cumulative hypoxic exposure. As Gangaram-Panday et al. have recently emphasized, substantial variations in desaturation definitions, thresholds, and monitoring strategies can affect the detection and interpretation of hypoxemia, which complicates the comparison of findings across clinical studies [21].

The dissociation between frequency and depth underlines a physiological mechanism centered on improved oxygen-carrying capacity rather than altered respiratory drive or airway stability [13]. The extent of the transfusion benefit may be scaled with baseline respiratory stability. Poppe et al. found stronger effects in infants with higher desaturation frequency [4]. Kovatis et al. reported improvements mainly during more severe events (<75/80%), although their inclusion of all types of ventilatory support in their study population limits comparability to our exclusively non-intubated cohort [13]. Cibulskis et al. demonstrated that transfusions increased systemic oxygen delivery by raising hemoglobin levels, which may reduce tissue hypoxia even in the absence of changes in SpO_2_ or ventilatory parameters [3]. Zheng et al. supported this by showing that transfusion improved cerebral and splanchnic oxygenation, despite stable global measures such as FiO_2_ or heart rate [22]. Together, these findings support the interpretation that transfusion may mitigate the severity of hypoxic episodes through enhanced local oxygenation, rather than preventing their occurrence. The secondary analysis of the ETTNO trial indicates that even a liberal transfusion strategy did not mitigate the occurrence of intermittent hypoxemia. This suggests that increasing systemic oxygen-carrying capacity does not address the underlying immature respiratory regulation that precipitates these events [23].

We observed no significant change in baseline heart rate, consistent with Fredrickson et al., who reported decreases only in severely anemic infants [14]. This suggests that the systemic responses to transfusions may depend on baseline hematologic status. In our clinically stable infants, transfused according to restrictive institutional thresholds, autonomic compensation may have been sufficient to mitigate the quantifiable effects on heart rate.

FiO_2_ requirements remained unchanged post-transfusion, aligning with Kovatis et al., who also reported this in their mixed population [13]. The unchanged FiO_2_ requirement, together with the stable frequency of interventions in response to bradycardia or desaturation episodes, supports the interpretation that while transfusion may alleviate the severity of individual events, it does not substantially reduce the need for clinical responses in this population.

While the present study offers meaningful insight into the short-term physiological effects of transfusion in VLBW infants, several limitations should be acknowledged. The retrospective design limits causal inference, and the possibility of confounding by indication cannot be excluded, as less stable infants may have been more likely to receive transfusions. This raises the possibility of reverse causality, whereby observed improvements may reflect spontaneous stabilization rather than transfusion effects. The short observation period helps mitigate maturational effects, although residual influences cannot be ruled out. Intervention-related data (stimulation events) relied on manual documentation and were not classified by type or duration. We did not account for postnatal age, despite evidence indicating that hypoxic episodes increase during the first weeks of life [18,24,25]. Age-dependent analyses could clarify whether transfusion effects differ between early and later postnatal phases, an area that warrants prospective study. As indicated by extant literature, the effects of transfusions may vary depending on the age of the patient; older preterm infants may demonstrate a greater degree of benefit [18,26]. Apneic episodes, which likely precede both desaturations and bradycardias, were not assessed. Likewise, cumulative hypoxic burden (e.g., area under the SpO_2_ curve) was not considered. Data on differences in pre- and post-transfusion hemoglobin levels were not included in the analysis. Moreover, the analysis did not evaluate modifications in respiratory support settings or transitions between NIPPV, CPAP and spontaneous breathing. Subgroup comparisons by support modality were not conducted, which may limit the applicability of the findings to more heterogeneous clinical populations. The lower margin (90%) of the target saturation range might be considered a potential limitation, as it results in a higher number of desaturation episodes compared to a lower saturation target [20]. However, as the post-transfusion desaturations were less pronounced, a lower target range would discriminate even better between the pre- and post-transfusion group regarding desaturation episodes; therefore, the saturation target does not weaken the results of the study regarding the reduced number of desaturation episodes following PRBC transfusion. Despite these limitations, the study benefits from a clearly defined patient population, continuous electronic monitoring, and a within-subject design. These elements, when considered collectively, provide substantial insight into the short-term transfusion effects in this group of preterm infants.

## 5. Conclusions

In clinically stable very-low-birth-weight infants receiving non-invasive ventilatory support, packed red blood cell transfusion is associated with modest, short-term improvements in cardiorespiratory stability. However, these effects are limited in scope. Further research is needed to identify which patient subgroups derive the most significant improvement from these transfusions.

## Figures and Tables

**Table 1:** Baseline characteristics of VLBW infants included in the final analysis (*n* = 68).

	Study Population (*n* = 68)
Birth weight (kg, median, IQR, range)	0.72 (IQR 0.59 to 0.91, range 0.36 to 1.45)
Gestational age (weeks, median, IQR, range)	26 + 1 (IQR 25 + 1 to 27 + 5, range 23 + 5 to 34 + 0)
LOS (days, median, IQR, range)	93 (IQR 72 to 104, range 29 to 154)
SGA (*n*, %)	26 (38.2%)
Multiple gestation (*n*, %)	35 (51.5%)
Female (*n*, %)	25 (36.8%)
Delivery mode C-section (*n*, %)	63 (92.6%)
Sepsis/infection (*n*, %)	52 (76.5%)
IVH (*n*, %)	10 (14.7%)
BPD (*n*, %)	18 (26.5%)
NEC (*n*, %)	10 (14.7%)

Data represent patient demographics and clinical parameters documented during the entire neonatal intensive care unit stay. Legend: IQR, interquartile range; LOS, length of stay; SGA, small for gestational age; IVH, intraventricular hemorrhage (grade 2 & 3); BPD, bronchopulmonary dysplasia; NEC, necrotizing enterocolitis.

## Data Availability

The data presented in this study are not publicly available due to institutional regulations and the need to protect patient confidentiality. Requests to access the datasets may be directed to the corresponding author and will be considered in accordance with institutional policies and applicable data protection regulations.

## Abbreviations

The following abbreviations are used in this manuscript:

VLBW	Very low birth weight
PRBC	Packed red blood cell
NICU	Neonatal intensive care unit
CPAP	Continuous positive airway pressure
NIPPV	Noninvasive positive pressure ventilation
bpm	Beats per minute
SpO_2_	Peripheral oxygen saturation
FiO_2_	Fraction of inspired oxygen
ECG	Electrocardiography
